# White rice consumption and risk of esophageal cancer in Xinjiang Uyghur Autonomous Region, northwest China: a case-control study

**DOI:** 10.1186/s41043-015-0019-8

**Published:** 2015-07-10

**Authors:** Li Tang, Fenglian Xu, Taotao Zhang, Jun Lei, Colin W. Binns, Andy H. Lee

**Affiliations:** 1 School of Public Health, Curtin University, GPO Box U 1987, Perth, WA 6845 Australia; 2 National Drug and Alcohol Research Centre, University of New South Wales, Sydney, NSW Australia; 3 School of Medicine, Shihezi University, Shihezi, Xinjiang China; 4 Xinjiang Tumour Hospital, Urumqi, Xinjiang China

**Keywords:** Case-control study, White rice, Esophageal cancer, Xinjiang, China

## Abstract

This study investigated the association between white rice consumption and the risk of esophageal cancer in remote northwest China, where the cancer incidence is known to be high. A case-control study was conducted during 2008–2009 in Urumqi and Shihezi, Xinjiang Uyghur Autonomous Region of China. Participants were 359 incident esophageal cancer patients and 380 hospital-based controls. Information on habitual white rice consumption was obtained by personal interview using a validated semi-quantitative food frequency questionnaire. Logistic regression analyses were performed to assess the association between white rice consumption and the esophageal cancer risk. Confounding variables including socio-demographics, family history, dietary and lifestyle factors were adjusted in the multivariate model. The esophageal cancer patients reported lower consumption levels of white rice-based products, including cooked white rice and porridge, when compared to the control group. Overall, regular consumption of white rice foods was inversely associated with the esophageal cancer risk, the adjusted OR being 0.34 (95 % CI 0.23 to 0.52) for the highest (>250 g) versus the lowest (<92 g) tertile of daily intake. Similar reductions in risk were also apparent for high consumption levels of cooked white rice and porridge. In conclusion, habitual white rice consumption was associated with a reduced risk of esophageal cancer for adults residing in northwest China. Our findings provide evidence to support the continued consumption of white rice.

## Background

Globally, esophageal cancer is the eighth most common malignancy and the sixth leading cause of cancer-related deaths. In 2008, more than 480,000 new cases were diagnosed and approximately 407,000 deaths were attributable to this disease [1]. The incidence rates of esophageal cancer vary substantially between countries and regions, which suggest that dietary and lifestyle factors may play an important role in its aetiology [2].

Rice is a staple food for more than half of the world’s population [3]. White rice, produced through a series of refining processes, is the predominant type of rice consumed in Asian countries [4]. A few studies have found that the consumption of white rice may be linked to the development of type 2 diabetes [5, 6]. However, further substantive evidence is required to confirm its effect.

Xinjiang Uyghur Autonomous Region, located in the northwest of China, is one of the areas constituting the so-called ‘Asian Esophageal Cancer Belt’ [7]. Factors found to be consistently associated with an increased risk of esophageal cancer in this ‘cancer belt’ include a diet poor in fruit and vegetables intake, tobacco smoking and alcohol drinking [7]. According to a survey undertaken in Xinjiang between 2005 and 2008, the incidence of esophageal cancer was 30.2 per 100,000 adults, much higher than the national average of 19.3 per 100,000 [8]. White rice-based products, especially cooked white rice and porridge, are commonly consumed in China. In view of the lack of epidemiological data, the present study investigated the association between white rice consumption and the risk of esophageal cancer in this remote area of China.

## Methods

### Study design and participants

A hospital-based case-control study of esophageal cancer was conducted in Urumqi and Shihezi, Xinjiang Uyghur Autonomous Region of China, between January 2008 and December 2009. Subjects were recruited from Xinjiang Tumour Hospital, Shihezi People’s Hospital, Kuitong Hospital and No. 1 Affiliated Hospital of Shihezi University.

Medical records and pathology reports were searched to identify incident patients diagnosed within the past 12 months. Pathological diagnoses were based on the International Classification of Disease for Oncology (ICD-O-3 codes: C150-C155, C158, C159) [9]. Patients without histopathological confirmation and those reported memory problems were excluded. Of the total 364 incident patients identified, 359 consented to participate in the study.

Controls were recruited from inpatient wards at the same hospitals from the Departments of Ophthalmology, Orthopedic, Respiratory Disease and Physiotherapy. Exclusion criteria for controls were previous diagnosis of any malignant disease, on long-term medical diet, and self-reported memory problems. Whenever more controls were available than could be interviewed, the final selection was made using random numbers. Of the 400 eligible controls recruited to frequency matched with cases by gender and age (within 5 years), 380 eventually gave their consent to be interviewed. No significant differences in age, gender and demographics were found between participants and non-participants.

The study protocol was approved by the participating hospitals and the Human Research Ethics Committee of Curtin University (approval number HR 56/2006), and conformed to the provisions of the Declaration of Helsinki. Written informed consent was obtained from all participants, who were assured of confidentiality of the information provided and their right to withdraw at any time without prejudice.

### Data collection

All participants were interviewed personally by trained hospital staff, usually in the presence of their next-of-kin to help the recall of past events. The structured questionnaire used composed sections on demographic characteristics, anthropometry, past and family medical history, diet, and lifestyle such as cigarette smoking and alcohol drinking. Information on dietary habits was collected using a 137-item semi-quantitative food frequency questionnaire which had been validated in both Han and ethnic minority groups [10]. The questionnaire included fruits, vegetables, meat and white rice products commonly consumed in northwest China. Frequency and amount of intake were recorded in detail. The reference recall period for dietary variables was set at 5 years before diagnosis for cases and 5 years before interview for controls. The energy content of each food or beverage item was obtained from the Chinese Food Composition Tables to calculate total energy intake (kcal/day) [11].

### Statistical analysis

Chi-square and t tests were used to compare the sample characteristics between case and control groups. Total white rice intake (g/day) was defined as the sum of daily consumption of cooked white rice, porridge and glutinous rice. For each exposure variable of interest, the tertiles corresponding to the distribution of controls were used to derive the cutoff points, resulting in three increasing levels of exposure, with the lowest level of intake being the reference category. However, glutinous rice intake was categorised as a binary variable due to its low and infrequent consumption by the Xinjiang adults.

Both crude and adjusted odds ratio (OR) and corresponding 95 % confidence interval (CI) were computed using unconditional logistic regression analyses. Confounding variables included in the separate models were age (years), gender, education level (none/primary, secondary, tertiary), annual income (<5000 yuan, 5000–20,000 yuan, > 20,000 yuan), body mass index (5 years ago, kg/m^2^), vegetable consumption (g/day), fruit consumption (g/day), meat consumption (g/day), total energy intake (kcal/day), smoking status (never, ever), alcohol drinking (never/seldom, often) and family history of cancer in first-degree relatives (no, yes). These variables were either established or plausible risk factors from the literature. All statistical analyses were performed in the SPSS package version 20.

## Results

Table 1 summarises characteristics of the sample by case-control status. The participants were on average 61 (SD 11.4) years old with mean body mass index 24.1 (SD 3.7) kg/m^2^. Most (about 72 %) of them were male. About half the participants were smokers and regularly drank alcoholic beverages. Compared to the controls, patients with esophageal cancer tended to belong to the ethnic minority group, have lower education level but a family history of esophageal cancer, have a marginally lower daily energy intake, and consume significantly less vegetables and fruits in daily life. With respect to white rice-based products, the cases reported lower levels of cooked rice and porridge intake than the controls, whereas less than half the participants ate glutinous rice on a daily basis for both groups.

**Table 1:** Comparison of demographic factors and white rice consumption between case and control groups in northwest China

Variable	Cases	Controls	*p* ^a^
	n (%)	n (%)	
Gender			0.623
Male	260 (72.4 %)	269 (70.8 %)	
Female	99 (27.6 %)	111 (29.2 %)	
Ethnic group			0.001
Han	270 (75.2 %)	322 (84.7 %)	
Minority	89 (24.8 %)	58 (15.3 %)	
Education level			<0.001
None/primary	183 (51.0 %)	136 (35.8 %)	
Secondary	140 (39.0 %)	191 (50.3 %)	
Tertiary	36 (10.0 %)	53 (13.9 %)	
Annual income (yuan^b^)			0.112
<5000	130 (36.2 %)	132 (34.7 %)	
5000–20,000	177 (49.3 %)	171 (45.0 %)	
>20,000	52 (14.5 %)	77 (20.3 %)	
Smoking status			0.188
Never	164 (45.7 %)	192 (50.5 %)	
Ever	195 (54.3 %)	188 (49.5 %)	
Alcohol drinking			0.216
Never/seldom	193 (53.8 %)	187 (49.2 %)	
Often	166 (46.2 %)	193 (50.8 %)	
Family history of cancer in first-degree relatives			<0.001
No	306 (85.2 %)	356 (93.7 %)	
Yes	53 (14.8 %)	24 (6.3 %)	
Age at interview (years): mean (SD)	61.4 (11.0)	60.6 (11.8)	0.338
BMI (5 years ago, kg/m^2^): mean (SD)	24.3 (3.8)	24.0 (3.6)	0.181
Vegetable consumption (g/day): mean (SD)	677.8 (542.6)	874.3 (621.8)	<0.001
Fruit consumption (g/day): mean (SD)	342.0 (410.6)	463.1 (480.6)	<0.001
Meat consumption (g/day): mean (SD)	232.1 (263.1)	242.3 (264.1)	0.601
Energy intake (kcal/day): mean (SD)	4310 (2681)	4709 (2716)	0.047
Total white rice (g/day): mean (SD)	137.8 (188.0)	208.7 (208.9)	<0.001
Cooked white rice (g/day): mean (SD)	79.5 (133.1)	111.2 (132.9)	0.001
Porridge (g/day): mean (SD)	53.0 (83.9)	95.2 (148.3)	<0.001
Glutinous rice	157 (43.7 %)	183 (48.2 %)	0.228

^a^Chi-square or *t*-test for difference between cases and controls

^b^1 yuan ≅ 0.16 USD

Table 2 presents the results of logistic regression analyses. Overall, regular consumption of white rice foods was inversely associated with the esophageal cancer risk, and the dose-response relationship was significant (*p* for trend < 0.001). The adjusted OR was 0.34 (95 % CI 0.23 to 0.52) for adults consuming over 250 g relative to those less than 92 g per day. Similar reductions in cancer risk were also apparent for high consumption levels of cooked white rice and porridge, but not from eating glutinous rice. Further subgroup analysis by ethnicity (Han versus Uyghur minority people) produced similar results which were omitted for brevity.

**Table 2:** Crude and adjusted odds ratio (95 % confidence interval) of esophageal cancer risk for white rice consumption in northwest China

Daily intake (g)	Cases	Controls	Crude OR	Adjusted OR^a^
	n (%)	n (%)	(95 % CI)	(95 % CI)
Total white rice				
<92	195 (54.3 %)	125 (32.9 %)	1.00	1.00
92–250	101 (28.1 %)	130 (34.2 %)	0.50 (0.35, 0.70)	0.51 (0.36, 0.74)
>250	63 (17.5 %)	125 (32.9 %)	0.32 (0.22, 0.47)	0.34 (0.23, 0.52)
Cooked white rice				
<30	187 (52.1 %)	128 (33.7 %)	1.00	1.00
30–140	95 (26.5 %)	120 (31.6 %)	0.54 (0.38, 0.77)	0.64 (0.44, 0.92)
>140	77 (21.4 %)	132 (34.7 %)	0.40 (0.28, 0.57)	0.48 (0.33, 0.71)
Porridge				
<28	194 (54.0 %)	124 (32.6 %)	1.00	1.00
28–100	106 (29.5 %)	136 (35.8 %)	0.50 (0.36, 0.70)	0.52 (0.36, 0.75)
>100	59 (16.4 %)	120 (31.6 %)	0.31 (0.21, 0.46)	0.34 (0.22, 0.50)
Glutinous rice				
no	202 (56.3 %)	197 (51.8 %)	1.00	1.00
yes	157 (43.7 %)	183 (48.2 %)	0.84 (0.63, 1.12)	0.93 (0.68, 1.28)

^a^From separate logistic regression models adjusting for age (years), gender, education level (none/primary, secondary, tertiary), annual income (<5000 yuan, 5000–20,000 yuan, > 20,000 yuan), body mass index (5 years ago, kg/m^2^), vegetable consumption (g/day), fruit consumption (g/day), meat consumption (g/day), total energy intake (kcal/day), smoking status (never, ever), alcohol drinking (never/seldom, often) and family history of cancer in first-degree relatives (no, yes)

## Discussion

The present study provided the first report on the inverse association between habitual white rice consumption and esophageal cancer risk. Our results were somewhat different from a previous study undertaken in India which observed similar frequency of rice consumption between esophageal cancer patients and controls [12]. Despite the lack of a comprehensive biological mechanism underlying the relationship between white rice and esophageal cancer development, an experimental study has demonstrated the anti-carcinogenic property of white rice through its growth-inhibiting and immunopotentiating effects on leukemic cells [13]. Even after the refining process, white rice still contains antioxidant nutrients and essential amino acids which are required for a good health [14].

In this study, a standardised identification procedure had been implemented that ensured the ascertainment of cases was maximised and complete. To avoid misclassification of the case-control status, we recruited only incident patients who had been diagnosed with esophageal cancer within the past 12 months and subsequently confirmed with pathology. All controls were carefully screened. It was possible that some esophageal cancer patients might have modified their dietary behaviours since the onset of the disease. Therefore, the reference period for the dietary recall was set at 5 years before diagnosis to minimise reverse causation.

Several other issues should be considered when interpreting the findings. The use of hospital-based controls may lead to Berksonian bias if their characteristics are different from those of the general population. Four hospitals serving the entire catchment region were used for recruitment to reduce the selection bias. Although the recall of habitual white rice consumption should not be affected by the case-control status, dietary assessment was made based on self-report, which probably introduced some recall error in the response of participants, especially since the recall period of dietary intakes was set at 5 years ago. Face-to-face interviews were thus arranged in the presence of next-of-kin to help improve the accuracy of their responses. Information bias and recall bias were unlikely because all participants remained blind to the study hypothesis, while the protective role of white rice has not yet been established. It may be argued that white rice consumption is a marker of an (unidentified) healthy lifestyle against esophagus disease development. Therefore, plausible demographic and lifestyle confounding factors, including fruit and vegetables consumption, were adjusted for in the logistic regression analyses. Nevertheless, the possibility of residual confounding effects could not be ruled out. Finally, future studies with information on the pathologic severity of esophageal cancer at the time of diagnosis are recommended to enable a more comprehensive profile of the patients.

## Conclusions

In conclusion, inverse associations were observed between white rice products and the esophageal cancer incidence among Xinjiang adults. Further prospective cohort studies in this high risk area of China and elsewhere are required to confirm the effects of long term consumption.
